# Field-based remote sensing models predict radiation use efficiency in wheat

**DOI:** 10.1093/jxb/erab115

**Published:** 2021-05-04

**Authors:** Carlos A Robles-Zazueta, Gemma Molero, Francisco Pinto, M John Foulkes, Matthew P Reynolds, Erik H Murchie

**Affiliations:** 1 Division of Plant and Crop Sciences, School of Biosciences, University of Nottingham, Sutton Bonington Campus, Leicestershire LE12 5RD,UK; 2 International Maize and Wheat Improvement Center (CIMMYT), carretera Mexico-Veracruz km 45, El Batan, Texcoco, Mexico CP; 3 KWS Momont Recherche, 7 rue de Martinval, 59246 Mons-en-Pevele,France

**Keywords:** High-throughput phenotyping, hyperspectral reflectance, partial least squares regression, physiological breeding, RUE, vegetation indices, wheat

## Abstract

Wheat yields are stagnating or declining in many regions, requiring efforts to improve the light conversion efficiency, known as radiation use efficiency (RUE). RUE is a key trait in plant physiology because it links light capture and primary metabolism with biomass accumulation and yield, but its measurement is time consuming and this has limited its use in fundamental research and large-scale physiological breeding. In this study, high-throughput plant phenotyping (HTPP) approaches were used among a population of field-grown wheat with variation in RUE and photosynthetic traits to build predictive models of RUE, biomass, and intercepted photosynthetically active radiation (IPAR). Three approaches were used: best combination of sensors; canopy vegetation indices; and partial least squares regression. The use of remote sensing models predicted RUE with up to 70% accuracy compared with ground truth data. Water indices and canopy greenness indices [normalized difference vegetation index (NDVI), enhanced vegetation index (EVI)] are the better option to predict RUE, biomass, and IPAR, and indices related to gas exchange, non-photochemical quenching [photochemical reflectance index (PRI)] and senescence [structural-insensitive pigment index (SIPI)] are better predictors for these traits at the vegetative and grain-filling stages, respectively. These models will be instrumental to explain canopy processes, improve crop growth and yield modelling, and potentially be used to predict RUE in different crops or ecosystems.

## Teaser

Radiation use efficiency can be predicted with ~70% accuracy. Canopy water content, greenness, and gas exchange spectral indices are the best predictors for RUE, biomass accumulation, and light interception.

## Introduction

Staple crop yields must increase by at least at a rate of 2.4% per year to ensure food security for a growing population, dietary changes, and expanding use of biofuels (Foley *et al.*, 2011; Ray *et al.*, 2013). Recent studies suggest that yield gains for staple crops are on average 1.2–1.3% year^−1^; therefore, it will not be sufficient to meet 2050 food demands (Ray *et al.*, 2012). Moreover, climate change predictions of future environmental conditions suggest that crops will be subjected to higher temperatures, flooding, drought, and shifts in precipitation patterns, which will affect development, primary metabolic events, biomass accumulation, and yield (Porter *et al.*, 2014; Asseng *et al.*, 2015; Garatuza-Payan *et al.*, 2018). The socio-economic repercussions will be felt worldwide, but mostly in low-income countries (Rajaram *et al.*, 1993), and the challenge of raising staple crop yields is one of the main goals for the scientific community in this century (Bailey-Serres *et al.*, 2019).

Wheat (*Triticum aestivum* L.) is one of the most important staple crops and its annual production is estimated to be ~770 Mt year^−1^ (Food and Agriculatural Organization of the United Nations, 2020). Physiological approaches for wheat improvement have had a pivotal role in reducing the gap between field and theoretical yields. So far, the main physiological traits improved in wheat have been reduction in plant height to minimize lodging, the partitioning of biomass into the grain, and optimization of leaf area index (LAI) (Foulkes *et al.*, 2011; Parry *et al.*, 2011; Reynolds *et al.*, 2012). It has been proposed that to further increase yield, it will be necessary to improve photosynthesis and the conversion rate of photosynthetically active radiation (PAR) to biomass by the canopy (Long *et al.*, 2006; Murchie *et al.*, 2009, 2018; Zhu *et al.*, 2010). This conversion rate is known as radiation use efficiency (RUE) and is defined as the biomass (dry weight) accumulated per unit of absorbed radiation, (g MJ^−1^) (Monteith, 1977).

Under yield potential conditions, yield has been defined as a function of incident PAR, the fraction of intercepted radiation during the crop cycle (FPAR, ε), RUE, and the relationship between grain dry weight and aboveground dry biomass [harvest index (HI)] (Reynolds *et al.*, 2005). This is expressed in Equation 1:

$$\begin{array}{l} Yield=\underset{i=1}{\overset{n}{\sum }}\;PAR_{i}\times FPAR_{i}\times RUE_{t}\times HI \end{array}$$ (1)

Where *n* is the day when a genotype reaches physiological maturity, PAR_*i*_ is the incident radiation on the *i*th day, FPAR_*i*_ the fraction of incident radiation absorbed on the *it*h day, and RUE_*t*_ the radiation use efficiency of the crop cycle.

Theoretically, yield could be improved by increasing any of the elements from Equation 1 but, since traits related to HI and light interception are close to optimization, the focus to increase yield should be shifted on improving RUE (Amthor, 2010; Zhu *et al.*, 2010; Parry *et al.*, 2011). Due to the complexity of RUE being the product of many underlying processes that are sensitive to the environment and the fact that measuring it is labour and cost intensive, its potential for increasing yield is not currently exploited in wheat breeding programmes. Therefore, it is necessary to develop high-throughput methods to measure and predict RUE for field research and breeding purposes.

The importance of RUE in plant physiology resides in the association of RUE and yield, as RUE alone can explain ~40% of its variability and it can help us to elucidate the roles of light capture and key plant processes of leaf biochemistry that drive biomass and yield (Hubbart *et al.*, 2018; Molero *et al.*, 2019). Evidence from FACE (free air CO_2_ enrichment) experiments suggests that there is room for RUE improvement that could be driven by leaf photosynthesis (Ainsworth and Long, 2005, 2020) and it has been suggested that even small increases in these two traits will have a major impact on wheat yield if HI can be maintained at modern level rates (Parry *et al.*, 2011). In contrast to the negative correlation existing between aboveground biomass measured at different growth stages and HI (Aisawi *et al.*, 2015; Molero *et al.*, 2019; Sierra-Gonzalez *et al.*, 2021), no negative correlations were observed between HI and RUE measured in the vegetative stages and across the whole crop cycle (Molero *et al.*, 2019). Hence, increasing RUE is a promising strategy to achieve further genetic gains in yield.

In order to measure RUE in a crop canopy, it is necessary to harvest aboveground biomass for at least two points in time, which is time consuming and can compromise the accuracy of yield measurements in the remaining plot area. In particular, if several harvests throughout the crop cycle are needed, this becomes a big issue for breeding programmes. However, this may be solved using non-invasive phenotyping techniques.

High-throughput phenotyping (HTPP) refers to the use of novel non-invasive techniques to measure physiological and agronomical traits (e.g. plant growth, biomass accumulation, gas exchange, canopy architecture, organ stoichiometry, and grain yield) combining multidisciplinary knowledge that allows plant phenotyping at different spatio-temporal (seconds to years) and hierarchical scales (cells to canopies) (Furbank and Tester, 2011; Fiorani and Schurr, 2013; Tardieu *et al.*, 2017; Araus *et al.*, 2018; Reynolds *et al.*, 2020).

Optical remote sensing techniques are among the most widely used for HTPP. These data usually range from 350 nm to 2500 nm, encompassing areas of the visible (PAR, 400–700 nm), near infrared (NIR, 700–1350 nm), red edge (680–730 nm), and shortwave infrared (SWIR; 1350–2500 nm) spectrum (Gamon *et al.*, 2019). Hyperspectral data have been used mainly in two ways: spectral indices (also known as vegetation indices, VIs) calculated from relationships between reflectance at specific wavelengths and physiological traits (Peñuelas *et al.*, 1997; Blackburn, 1998; Cabrera-Bosquet *et al.*, 2011; Tattaris *et al.*, 2016); and by using the whole spectra as an individual data point to predict traits of interest (e.g. leaf N and C content, CO_2_ assimilation, respiration, maximum velocity of Rubisco carboxylation, electron transport rate, leaf mass, and specific leaf areas) using statistical methods such as partial least squares regression (PLSR) (Serbin *et al.*, 2014; Silva-Pérez *et al.*, 2017; Yendrek *et al.*, 2017; Coast *et al.*, 2019; Fu *et al.*, 2020). The advantage of these two approaches is that hundreds or thousands of lines can be screened without the need for destructive and time-consuming field sampling. Moreover, as HTPP technologies become cheaper, crop physiologists and breeders will be able to study complex traits more cost effectively (Reynolds *et al.*, 2020).

Previous studies have predicted yield in wheat and rye (Montesinos-López *et al.*, 2017; Galán *et al.*, 2020), aboveground biomass in wheat, rice, rye, and barley (Babar *et al.*, 2006; Gnyp *et al.*, 2014; Marshall and Thenkabail, 2015; Galán *et al.*, 2020), and RUE in maize (Tewes and Schellberg, 2018) using optical remote sensing approaches; however, to date, there is not such an effort to predict RUE in the field using a HTPP physiological breeding approach for wheat. The impact of predicting a multicomponent trait such as RUE with a HTPP approach in field conditions would be very high for physiological breeding programmes, while its full implementation would be very feasible in the medium term (Furbank *et al.*, 2019; Roitsch *et al.*, 2019).

Our hypothesis is that prediction models using canopy reflectance data will be more accurate than models using a different combination of sensors (which include leaf reflectance), due to a better representation of canopy processes. The objectives of this study are the prediction of RUE, biomass, and intercepted PAR (IPAR) with HTPP techniques based on VIs and PLSR models to define which approach will help more to alleviate the phenotyping bottleneck of these traits.

## Materials and methods

### Wheat population

Spring bread wheat cultivars were chosen from the ‘Photosynthesis Respiration Tails’ (PS Tails) trial which consisted of 80 genotypes including advanced line material coming from the High Biomass Association Panel (HiBAP) from CIMMYT. This germplasm is characterized by their high aboveground biomass and for containing lines with contrasting RUE expression that varies from the vegetative to the grain-filling phase, and has breeding value as it represents material that breeders use for their crosses for yield potential (for further information on HiBAP, see Molero *et al.*, 2019). For this study, a subset of 11 genotypes (Table 1) was selected based on RUE at the vegetative and grain-filling stages, yield, HI, flag leaf photosynthesis, and plant height to consider different levels of productivity and contrasting canopy architecture. This selection was made using data available from the 2016/2017 field season at CIMMYT’s experimental station.

**Table 1.** Reference ID, cross name, average days to initiation of booting (DTInB), days to anthesis (DTA), days to physiological maturity (DTPM), intercepted PAR (MJ), aboveground biomass (g m–2), and radiation use efficiency (g MJ–1) measured at different growth stages for the wheat genotypes studied

ID	Cross name	DTInB	DTA	DTPM	IPARE40	IPARInB	IPARA7	IPARPM	BME40	BMInB	BMA7	BMPM	RUE_E40InB	RUE_InBA7	RUE_preGF	RUE_GF	RUE_Total
1	KRICHAUFF^*a*^	60	77	119	224.39	365.82	543.27	833.56	188.47	466.32	978.38	1348.94	1.75	2.91	2.33	1.21	1.61
2	W15.92/4/PASTOR//HXL7573/2*BAU/3/WBLL1	59	74	113	230.22	359.2	524.44	762.38	217.14	515.19	905.55	1210.31	2.21	2.3	2.11	1.26	1.55
3	KUKRI	64	79	117	232.446	405.17	575.98	836.45	196.14	569.52	1075.3	1319.4	2.06	3.08	2.52	1.04	1.62
4	MUNAL #1	65	80	116	226.94	406.3	565.17	804.02	199.21	558.62	998.14	1235.51	1.87	2.8	2.27	0.85	1.51
5	JANZ^*a*^	60	73	116	229	371.49	534.41	822.75	190.05	497.99	893.31	1260.97	2.08	2.39	2.21	1.41	1.57
6	CHEWINK #1	62	80	118	233.53	385.28	567.81	843.43	186.36	517.53	994.67	1319.23	2.02	2.57	2.32	1.12	1.62
7	SOKOLL//PUB94.15.1.12/WBLL1	60	75	116	232.73	375.19	551.88	833.83	211.96	495.62	943.88	1390.22	1.92	2.58	2.28	1.55	1.77
8	PUB94.15.1.12/FRTL/5/CROC_1/AE.SQUARROSA(205)// BORL95/3/PRL/SARA//TSI/VEE#5/4/FRET2	59	74	116	230.92	368.04	538.45	824.21	214.35	551.04	1027.9	1445.18	2.26	2.71	2.56	1.43	1.8
9	C80.1/3*QT4118//KAUZ/RAYON/3/2*TRCH/7/CMH79A.955/4/ AGA/3/4*SN64/CNO67//INIA66/5/NAC/6/RIALTO /8/WBLL1*2/KURUKU	64	80	120	230.75	409	576.06	870.62	205.15	607.09	1127.2	1416.93	2.11	2.77	2.55	1.15	1.68
10	QUAIU*2/KINDE	58	74	114	223.5	346.8	522.34	759.87	206.70	517.04	987.58	1345.11	2.35	2.59	2.44	1.37	1.72
11	BORLAUG100 F2014^*a*^	59	74	115	228	357.51	525.58	791.4	202.45	444.28	953.7	1259.33	1.65	2.86	2.35	1.19	1.57
	Mean	61	76	116	229.31	377.26	547.76	816.59	201.64	521.84	989.59	1322.83	2.03	2.69	2.36	1.23	1.64
	H^2^	0.91	0.92	0.84	0	0.88	0.92	0.76	0	0.48	0.57	0.7	0.23	0	0.25	0.46	0.6
	G	***	***	***	ns	***	***	***	ns	ms	*	**	ns	ns	ns	ms	*
	Y	***	***	***	***	*	***	***	ns	ns	ns	**	*	ns	ns	ns	***
	G×Y	***	***	***	ns	*	ns	*	**	*	ns	*	ns	ns	ns	ns	ms

^*a*^ Genotypes studied only in Y2 and Y3.

BM_E40, biomass 40 d after emergence; BM_InB, biomass at initiation of booting; BM_A7, biomass 7 d after anthesis; BM_PM, biomass at physiological maturity; IPAR_E40, accumulated intercepted PAR 40 d after emergence; IPAR_InB, accumulated intercepted PAR at initiation of booting; IPAR_A7, accumulated intercepted PAR 7 d after anthesis; IPAR_PM, accumulated intercepted PAR at physiological maturity; RUE_E40InB, RUE from the period of 40 d after emergence to initiation of booting calculated with APAR; RUE_InBA7, RUE from the period of initiation of booting to 7 d after anthesis calculated with APAR; RUE_preGF, RUE pre-grain filling calculated with APAR; RUE_GF, RUE grain filling calculated with APAR; RUE_Total, RUE of the whole crop cycle calculated with APAR.

ms, marginally significant (0.1>*P*>0.05), *significant at *P*<0.05, **significant at *P*<0.01, ***significant at *P*<0.001, ns, not significant. H^2^=heritability, G=genotype, Y=environment, G×Y=genotype by environment interaction.

Eight genotypes were studied during the 2017/2018 field season, and the complete set of 11 lines were studied during the 2018/2019 and 2019/2020 field seasons (from now on referred to as Y1, Y2, and Y3, respectively). Experiments were carried out at CIMMYT’s Campo Experimental Norman E. Borlaug (CENEB) field station in Ciudad Obregon, Sonora, Mexico (27°23′46′′N, 109°55′42′′W, 38 m asl) during the spring wheat growth season that encompasses early December–early May.

### Field conditions

The experimental design was a randomized complete block design with three replications in raised beds, two beds per plot (bed width=0.8 m), and two rows per bed (row width=0.2 m) in 4 m×1.6 m plots in Y1 (plot area=6.4 m^2^). For Y2, the same experimental design was used but the number of replications was increased to four, and plot length increased to 5 m×1.6 m, increasing the area (plot area=8 m^2^). In Y3, the irrigation system was changed to optimize the water use and reduce lodging in the experimental station, and a drip irrigation system was put in place. A randomized complete block design was used with the same replications and plot area as Y2 but the plants were sown as six row plots with 15 cm between rows on the flat with drip irrigation.

Sowing dates were 5 December 2017, 6 December 2018, and 18 December 2019 for Y1, Y2, and Y3, respectively. Emergence dates were 12 December 2017, 12 December 2018, and 26 December 2019 (Y1, Y2, and Y3 respectively). Harvest dates were 8 May 2018, 30 April 2019, and 13 May 2020 (Y1, Y2, and Y3, respectively). Seed rate was ~250 g m^–2^ in the three years. Irrigation was applied four times during the crop cycle in ~25 d intervals [pre-sowing and 25, 50, 75, and 100 days after emergence (DAE)]. Plants were grown under optimal conditions in the field with pest control, weed control, and fertilization to avoid limitations to yield. In Y1, fertilization was applied in the form of urea (200 kg N ha^−1^) 25 DAE. For Y2, fertilization was divided into 100 kg N ha^−1^ 25 DAE and another 100 kg N ha^−1^ 58 DAE. Finally, for Y3, 100 kg N ha^−1^ were applied 30 DAE and 50 kg N ha^−1^ 50 DAE; 50 kg P ha^−1^ were applied in the three cycles when the first application of N was made.

Phenology was scored according to the Zadoks growth scale for cereals (Zadoks *et al.*, 1974). The growth stages recorded were initiation of booting (GS41), anthesis (GS65), and physiological maturity (GS87) when 50% of the shoots in the plot reached each stage. Meteorological data from a station near to the experimental site were collected for the whole crop cycle; thermal time and accumulated PAR were calculated for the growth stages where biomass was collected (Table 1).

In order to clarify the acronyms used in this study, the reader is referred to Table 4, where all abbreviations for phenological and ground truth traits are explained in detail.

**Table 4.** List of acronyms used in this study

Acronym	Meaning
BM_E40	Biomass harvested 40 d after emergence
BM_InB	Biomass harvested at initiation of booting (GS41)
BM_A7	Biomass harvested 7 d after anthesis (GS65 + 7 d)
BM_PM	Biomass harvested at physiological maturity (GS87)
IPAR_E40	Accumulated intercepted PAR 40 d after emergence
IPAR_InB	Accumulated intercepted PAR at initiation of booting (GS41)
IPAR_A7	Accumulated intercepted PAR 7 d after anthesis (GS65 + 7 d)
IPAR_PM	Accumulated intercepted PAR at physiological maturity (GS87)
RUE_E40InB	RUE from the period of 40 d after emergence to initiation of booting
RUE_InBA7	RUE from the period of initiation of booting to 7 d after anthesis
RUE_preGF	RUE from the period of 40 d after emergence to 7 d after anthesis (pre-grain filling)
RUE_GF	RUE from the period of 7 d after anthesis to physiological maturity (grain filling)
RUE_Total	RUE from the period of 40 d after emergence to physiological maturity (crop cycle)
FL	Measurement taken at the flag leaf
SL	Measurement taken at the second leaf
TL	Measurement taken at the third leaf
can	Measurement taken above the canopy
vg	Data averaged from the vegetative period (from canopy closure to 7 d after anthesis)
gf	Data averaged from the grain filling period (from 7 d after anthesis to late grain filling)

### Ground truth traits

#### Light interception

The percentage of light intercepted (LI) was measured using a linear ceptometer (AccuPAR LP-80, Decagon Devices, Pullman, WA, USA) at 40 DAE (canopy closure), GS41, and GS65 + 7 d. Incident, reflected, and transmitted PAR through the canopy were measured at around 11.00–13.00 h when clear skies and low wind velocity conditions prevailed following phenotyping protocols (Pask *et al.*, 2013). The following equation was used to calculate the percentage of LI by the canopy:

$$\begin{array}{l} LI\;\;\;\left(\;\%\;\right)=\frac{PAR_{i}-PAR_{r}-PAR_{g}}{PAR_{i}-PAR_{r}}\;\;\;\times \;\;\;100 \end{array}$$ (2)

where LI (%) is the percentage of light intercepted by the canopy, and PAR_i_, PAR_r_, and PAR_g_ are the incident, reflected and transmitted PAR, respectively.

LI (%) was used to estimate the amount of IPAR by the canopy in the same growth stages where aboveground biomass was harvested.

#### Aboveground biomass

Aboveground biomass was harvested at four key developmental growth stages: canopy closure (40 DAE), initiation of booting (GS41), initiation of the grain-filling period (GS65 + 7 d), and physiological maturity (GS87).

At 40 DAE, biomass was harvested in 0.4 m^2^ (25 cm for each bed in the plot) and, at GS41 and GS65 + 7 d, biomass was harvested in 0.8 m^2^ (50 cm for each bed in the plot). Biomass harvests were made leaving 25 cm (40 DAE) and 50 cm (GS41, GS65 + 7 d) at the northern side of the plots to reduce border effects in subsequent harvests. All fresh biomass was weighed, a subsample of 50 shoots was weighted and dried in an oven at 70 °C for 48 h, and dry weight was recorded. At GS87, biomass was calculated from the measurement of yield components. For every growth stage, the aboveground biomass was calculated as follows:

$$\begin{array}{l} Aboveground\;biomass=Subsample\;DW\times \frac{Total\;FW\times Harvested\;area}{Subsample\;FW} \end{array}$$ (3)

#### Radiation use efficiency

RUE was estimated from the slope of the linear regression between aboveground biomass and the corresponding accumulated IPAR during the determined growth period (Monteith, 1977). Incoming radiation from a nearby meteorological station was used to calculate the accumulated PAR by multiplying irradiance by 0.45 to convert it to PAR.

RUE observations in this study are presented for five different growth periods: canopy closure to GS41 (RUE_E40InB), GS41 to GS65 + 7 d (RUE_InBA7), pre-grain-filling stage (40 DAE to GS65 + 7 d, RUE_preGF), grain-filling stage (GS65 + 7 d to GS87, RUE_GF), and RUE of the crop cycle which comprises the period from canopy closure to physiological maturity (40 DAE to GS87, RUE_Total). For RUE_GF and RUE_Total, a correction factor was used to account for intercepted radiation during the last 25% (in days) of the grain-filling period when canopy leaves start to senesce based on a light interception model (Reynolds *et al.*, 2000). Calculations were made as follows:

$$\begin{array}{l} RUE\_E40InB=\frac{\left(BM\;GS41-BM\;E40\right)}{\left(Acc\;IPAR\;GS41-Acc\;IPAR\;E40\right)} \end{array}$$ (4)

$$\begin{array}{l} RUE\_InBA7=\frac{\left(BM\;GS65+7\;d-BM\;GS41\right)}{\left(Acc\;IPAR\;GS65+7\;d-Acc\;IPAR\;GS41\right)} \end{array}$$ (5)

$$\begin{array}{l} RUE\_preGF=\frac{\left(BM\;GS65+7\;\;\;d-BM\;E40\right)}{\left(Acc\;IPAR\;GS65+7\;d-Acc\;IPAR\;E40\right)} \end{array}$$ (6)

$$\begin{array}{l} RUE\_GF=\frac{\left(BM\;GS87-BM\;GS65+7\;d\right)}{\begin{array}{c} \left\{[(MJacc\_75\%\;grain\;f\;\;illing\right)\\ -[(Acc\;IPAR\;GS65+7d)\\ +(MJacc\_25\%\;grain\;f\;\;illing\times 0.5)]\} \end{array}} \end{array}$$ (7)

$$\begin{array}{l} RUE\_Total=\frac{\left(BM\;GS87-BM\;E40\right)}{\begin{array}{c} Acc\;IPAR\;GS87+[(MJacc\_25\%\;grain\;f\;\;illing\times 0.5)\\ -(Acc\;IPAR\;E40)]\;\;\; \end{array}} \end{array}$$ (8)

### Remote sensing measurements

Remote sensing data were collected above the canopy using a field spectroradiometer coupled with a pistol grip, and throughout the layers of the canopy (flag, second, and third leaves) with a field spectroradiometer coupled with a leaf clip. Chlorophyll content (SPAD) was measured in the flag, second, and third leaves with a SPAD-502 meter (Konika Minolta, Japan), canopy temperature (CT) was measured using an infrared thermometer (LT 300, Sixth Sense, USA), and the normalized difference vegetation index (NDVI) was measured using a Green Seeker (Trimble, USA) from canopy closure to late grain filling at least once a week as described by Pask *et al.* (2013).

Canopy reflectance was measured using a field spectroradiometer with a spectral range from 350 nm to 2500 nm with a 3 nm spectral resolution in the visible-near infrared (VNIR) and 10 nm resolution in the shortwave infrared (SWIR) spectrum equipped with an optic fibre with a field of view of 25° (ASD Field Spec® 3, Boulder, CO, USA). Reflectance was measured at 0.5 m at the nadir of the canopy with a pistol grip (ASD Field Spec® 3) under clear sky conditions and when low wind speeds were predominant to make sure we were collecting the signal from the canopy instead of soil or vegetation/soil mixed signals. Six data points were collected at each plot and then averaged to obtain the reflectance of each plot.

Leaf reflectance was measured using a leaf clip equipped with a halogen bulb as light source (ASD Field Spec® 3). Healthy leaves were clipped in the middle portion and measurements were taken for the flag, second, and third leaves in one fertile shoot per plot. The first measurement was taken at GS41 and the last at GS75. Both canopy and leaf reflectance data were averaged to obtain representative values from the vegetative period (40 DAE to GS55) and the grain-filling period (GS65 to GS75). Reflectance measurements were made between 10.00 h and 14.00 h where the sun is close to its zenith at this latitude.

For the electron transport rate (*J*_max_) and maximum velocity of Rubisco carboxylation/N content based on leaf area (*V*_cmax_/N_area_), leaf spectral data were used to predict them based on PLSR modelling using Wheat Physiology Predictor, a web tool developed to predict photosynthetic traits derived from light response and *A*–*C*i photosynthetic curves (www.metabolome-express.org/pheno/) (Silva-Pérez *et al.*, 2017).

### Data analysis

Adjusted means from each year were calculated for the ground truth and remote sensing traits as well as the predictions from PLSR using the linear model from package lme4 (R Studio) with the graphical user interface META-R v 6.04 (Alvarado *et al.*, 2020) as follows:

Yijk=μ+Re⁡pi+εijk (9)

Where Y*ijk* is the ground truth or remote sensing trait, μ is the mean effect, Rep*i* is the effect of the *i*th replicate, and ε*ijk* is the error associated with the *i*th replication.

If statistically significant differences were not found between genotypes, VIs were adjusted with phenology from GS41 for vegetative period averages and phenology from GS65 for grain-filling period averages as covariates. Phenotypic correlations between RUE and remote sensing traits (SPAD, CT, NDVI Green Seeker, and VIs) were calculated using Pearson product–moment correlations, and a threshold was established to select only VIs with statistically significant phenotypic correlations (*P*<0.05).

### Vegetation indices

After field sampling, average reflectance collected above the canopy and the leaves from each plot was processed using View Spec Pro software (Analytical Spectral Devices Inc., Boulder, CO, USA). These values were later used to calculate different VIs available from the literature (Li *et al.*, 2010; Garbulsky *et al.*, 2011; Ollinger, 2011; Pask *et al.*, 2013) and the Index Database (https://www.indexdatabase.de/) using R Studio (R Core Team, 2016). In Table 2, the VIs which correlated significantly with RUE, biomass, and IPAR, and were used for building the predictive models, are shown.

**Table 2.** Common remote sensing physiological traits found to correlate with radiation use efficiency, biomass, and intercepted PAR during the three field seasons measured in this study

Trait	Meaning	Equation	Physiological relevance	Reference
CT	Canopy temperature	N/A	Stomatal conductance, transpiration, root water uptake	Reynolds *et al.* (1994)
CRI	Carotenoid reflectance index	(1/R510)–(1/R550)	Carotenoid content	Steddom *et al.* (2003)
CUR	Curvature index	(R675×R690)/R683^2^	Diurnal variation of chlorophyll fluorescence, *F*_v_/*F*_m_	Zarco-Tejada *et al.* (2000)
EVI	Enhanced vegetation index	2.5[(R900–R680)/(R900 + 6×R680–7.5×R475 + 1)]	Photosynthetic capacity, canopy greenness without saturation problems	Huete *et al.* (2002)
GI	Green index	R554/R677	Canopy greenness, yield	Smith *et al.* (1995)
GNDVI-1	Green normalized differenced vegetation index-1	R810–[(R510+R561)/2]/R810+[(R510+R561)/2]	Canopy greenness, photosynthetic capacity, N status	Pask *et al.* (2013)
*J*_max_	Maximum electron transport rate	Partial least squares regression modelling	Leaf e^−^ transport rate	Silva-Pérez *et al.* (2017)
NDVI	Normalized differenced vegetation index	(R800–R680)/(R800+R680)	Chlorophyll content, canopy greenness, photosynthetic capacity, energy absorption	Tucker (1979)
NDVIGS	Normalized difference vegetation index measured with a Green Seeker sensor	(R800–R680)/(R800+R680)	Chlorophyll content, canopy greenness, photosynthetic capacity, energy absorption	Tucker (1979)
NDWI	Normalized difference water index	(R860–R1240)/(R860+R1240)	Canopy water content	Gao (1996)
NDWI-2	Normalized difference water index-2	(R970–R850)/(R970+R850)	Canopy water content	Babar *et al.* (2006)
NDWI-3	Normalized difference water index-3	(R970–R920)/(R970+R920)	Canopy water content	Babar *et al.* (2006)
NDWI-4	Normalized difference water index-4	(R970–R880)/(R970+R880)	Canopy water content	Babar *et al.* (2006)
NPCI	Normalized pigments chlorophyll ratio index	(R680–R430)/(R680+R430)	Canopy water and N status	Peñuelas *et al.* (1994)
OSAVI	Optimized soil-adjusted vegetation index	(1 + 0.16)(R800–R670)/(R800+R670 + 0.16)	Chlorophyll content and canopy greenness reducing the effect of soil interference	Daughtry *et al.* (2000)
PRI	Photochemical reflectance index	(R531–R570)/(R531+R570)	Carotenoid content, xanthopyll cycle, gas exchange, non-photochemical quenching	Peñuelas *et al.* (1995)
PSSRa	Pigment-specific simple ratio of chlorophyll *a*	R800/R675	Chl *a* content	Blackburn (1998)
PSSRb	Pigment-specific simple ratio of chlorophll *b*	R800/R650	Chl *b* content	Blackburn (1998)
RARSa	Ratio analysis of reflectance spectra of chlorophyll *a*	R675/R700	Chl *a* content	Chappelle *et al.* (1992)
RARSb	Ratio analysis of reflectance spectra of chlorophyll *b*	R675/(R650×R700)	Chl *b* content	Blackburn (1998)
RGR	Red green ratio	(R612+R660)/(R510+R560)	Red pigments and chlorophyll content	Steddom *et al.* (2003)
rNDVI	Red edge normalized difference vegetation index	(R750–R705)/(R750+R705)	Chlorophyll content, canopy greenness, photosynthetic capacity, energy absorption	Sims and Gamon (2002)
SAVI	Soil-adjusted vegetation index	[(R800–R680/R800+R680 + 0.75)](1 + 0.75)	Chlorophyll content and canopy greenness without soil interference	Huete (1988)
SIPI-1	Structure-insensitive pigment index-1	(R800–R445)/(R800–R680)	Carotenoid and chlorophyll content	Peñuelas *et al.* (1995)
SIPI-2	Structure-insensitive pigment index-2	(R800–R435)/(R415–R435)	Plant senescence related to stress	Pask *et al.* (2013)
SPAD	N/A	N/A	Plant chlorophyll content	Pask *et al.* (2013)
SR-1	Simple ratio-1	R800/R680	Canopy greenness and chlorophyll content	Sims and Gamon, (2002)
TCARI	Transformed chlorophyll absorption reflectance index	3[(R700–R670)–0.2(R700–R550)](R700/R670)	Canopy greenness, chlorophyll content, gas exchange reducing the effect of soil and non-photosynthetic components	Haboudane *et al.* (2002)
TCARI_705,750_	Transformed chlorophyll absorption reflectance index calculated with reflectance from 705 nm and 750 nm	3[(R750–R705)–0.2(R750–R550) (R750/R705)]	Canopy greenness, chlorophyll content, gas exchange reducing the effect of soil and non-photosynthetic components	Wu *et al.* (2008)
VARI	Visible atmospherically resistant index	(R560–R660)/(R560+R660-R459)	Canopy coverage	Steddom *et al.* (2003)
*V*_cmax_/N_area_	Maximum velocity of Rubisco carboxylation/N content based on leaf area	Partial least squares regression modelling	Photosynthetic N use efficiency	Silva-Pérez *et al.* (2017)
WI	Water index	R900/R970	Canopy water content	Peñuelas *et al.* (1997)

Vegetation indices were calculated with data collected with an ASD Field Spec hyperspectral radiometer and, when stated, Green Seeker sensors, an infrared thermometer, and a SPAD meter were also used to collect data.

### Partial least squares regression

Averaged reflectance spectral data of each plot collected above the canopy were post-processed to remove spurious data in areas of the spectra where negative or >1 values were present. Spectral reflectance from 350 nm to 1800 nm and from 1951 nm to 2450 nm were then used to predict RUE, biomass, and IPAR using the Principal Component and Partial Least Squares Regression package (pls) in R (Mevik and Wehrens, 2007) following the method proposed in Serbin *et al.* (2014).

While building the models, 80% of the dataset was used as training data and 20% was used as test data to validate the PLSR models. The number of components used in the models was based on the smallest root mean square error (RMSE) in the cross-validation stage (RMSEP-CV) and smallest prediction of the residual sum of squares (PRESS) from the training dataset. After these steps, PLSR modelling generates loadings and scores which are used to generate regression coefficients and intercepts for each individual wavelength, and thus the model can be built multiplying those values against each wavelength reflectance value. The regression coefficient (*R*^2^) and RMSE were considered to evaluate the three model approaches presented in this study. 

### Linear models

To build the linear models using the best combination of sensors (*bcs*) and VIs measured above the canopy (*cVI*), best subset regression was used with RUE, biomass, and IPAR of the different growth stages as dependent variables and the remote sensing traits as independent variables using the software Sigma Plot 13.0 (Systat Software Inc., San Jose, CA, USA). These linear models assume an association between the dependent and independent variables as follows:

$$\begin{array}{l} y=b_{0}+b_{1}x_{1}+b_{2}x_{2}+b_{3}x_{3}+\ldots b_{i}x_{i} \end{array}$$ (10)

Where *y* is the dependent variable, *x* the independent variable, and *b* the regression coefficients.

To compare the predictive ability of the linear models, a set of criteria was considered, such as the regression coefficient (*R*^2^), the variance inflation factor to avoid multicollinearity between the variables, and the RMSE, calculated as follows:

$$\begin{array}{l} RMSE=\;\;\;\sqrt{\frac{\overset{}{\sum }{\left(X_{i}-Y_{i}\right)}^{2}}{n}} \end{array}$$ (11)

Where *X*_*i*_ are the predicted values, *Y*_*i*_ the observed values, and *n* is the total number of observations.

## Results

### Accumulated IPAR

**Fig. 1.**
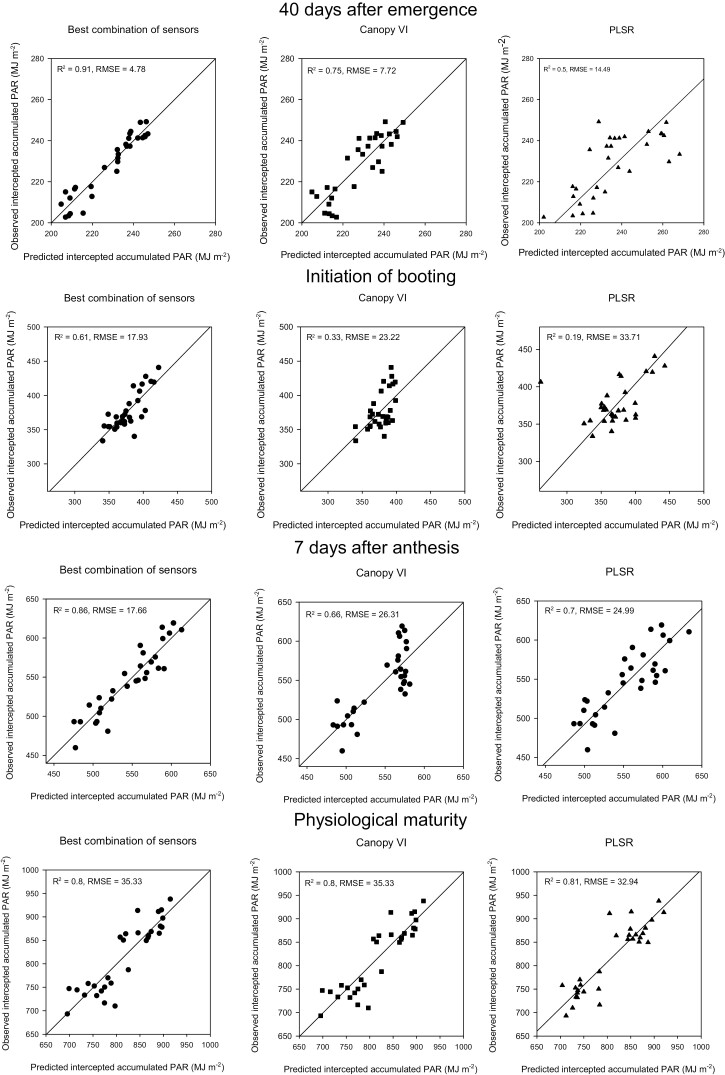
Intercepted accumulated PAR predictions with the different approaches used. Left panels represent predictions using the best combination of sensors (*bcs*), middle panels are predictions using vegetation indices derived from canopy reflectance (*cVI*), and the right panels represent predictions made with partial least squares regression (PLSR). Data points represent the genotype-adjusted means of the eight genotypes studied in Y1 and the 11 genotypes studied in Y2 and Y3 (*n*=30).

IPAR_E40_*bcs* was predicted using a linear combination of CTvg and NDVIGSvg. This model had the best performance of all methods and growth stages for this trait, with *R*^2^=0.91 and RMSE of 4.78 MJ m^−2^. In contrast, IPAR_E40_*cVI* was best predicted using NDWI-3 from the vegetative period, and model performance was *R*^2^=0.75 and RMSE of 7.72 MJ m^−2^. With PLSR modelling, the lowest *R*^2^=0.5 and highest prediction error RMSE=14.49 MJ m^−2^ were found for IPAR_E40 (Fig. 1).

IPAR_InB_*bcs* was predicted using NDVIGSvg and PRIvg measured at the canopy level. Model performance was the lowest for this trait, with *R*^2^=0.61 and RMSE=17.93 MJ m^−2^ (Table 3). IPAR_InB_*cVI* predictions were worse than IPAR_InB_*bcs*, but they were made only using the optimized soil-adjusted vegetation index (OSAVI) from the vegetative period with *R*^2^=0.33 and RMSE=23.22 MJ m^−2^. PLSR predictions were poor when the canopy was not fully closed, and we hypothesize that this could be due to mixed reflectance from leaves and soil affecting IPAR predictions with this method (Fig. 1).

**Table 3.** Models used to predict radiation use efficiency, biomass, and PAR interception at the different growth stages measured in this study

Trait	Model	*R* ^2^	Adj. *R*^2^	RMSE	*R* ^2^_bv	RMSE_bv
RUE_E40InB	–9.347 + 12.906WIcanvg–4.004NDVITLvg–0.795TCARITLvg	0.46	0.4	0.29	0.02	0.26
	–15.443–0.0674PSSRb_vg+16.469WI_vg	0.53	0.5	0.27	0.31	0.28
RUE_InBA7	–1.791 + 13.247NDWI-3canvg+4.721EVITLvg+6.656TCARI_705_TLvg	0.27	0.19	0.37	0.45	0.28
	7.543 + 28.717NDWI-3_vg–3.123EVI_vg	0.27	0.22	0.36	0.17	0.35
RUE_preGF	0.47 + 0.0446SPADTLvg	0.21	0.18	0.21	0.53	0.16
	19.762 + 0.0389CRI_vg–22.547NDVI_vg+10.455NDWI_vg+53.698PRI_vg	0.19	0.06	0.22	0.01	0.25
RUE_GF	–2.523–10.05VARIcanvg–4.661RARSacangf+16.258SIPI-1TLvg+1.17GITLgf–0.0112JFLgf – 0.0401V_cmax_/N_area_SLvg	0.61	0.51	0.23	0.55	0.18
	3.886–79.296PRI_vg–0.675GI_gf	0.27	0.22	0.29	0.01	0.36
RUE_Total	5.972–15.681NDWI-2canvg–5.458CURSLvg+2.21NPCITLgf	0.69	0.65	0.11	0.85	0.05
	0.845 + 0.992RGR_gf	0.53	0.51	0.13	0.23	0.15
BM_E40	294.202–0.394J_max_TLvg	0.2	0.17	24.53	0.01	31.07
	56.67 + 610.986WI_vg–844.888NDVI_vg+308.836SAVI_vg	0.17	0.07	25.83	0.09	31.4
BM_InB	89.423–220.49NDWI-4canvg+213.15GIFLvg–344.448TCARITLvg	0.42	0.35	53.35	0.09	56.14
	–206.393–7575.28NDWI-4_vg+737.072TCARI_vg	0.34	0.29	55.8	0.03	64.26
BM_A7	435.468 + 14412.02PRIcanvg+9039.943PRIFLgf	0.32	0.27	76.92	0.31	85.28
	696.304 + 15902.35PRI_vg	0.18	0.15	83.18	0.38	88.51
BM_PM	361.694 + 98.526PSSRaFLvg+106.66RARSbSLvg–1.52SIPI-2SLvg–135.394SR-1TLvg	0.67	0.62	74.39	0.38	77.12
	674.582–44.419CRI_vg+43.295PSSRa_vg–2.543SIPI2_vg	0.28	0.2	107.96	0.08	84.68
IPAR_E40	289.723–9.158CTvg+168.407NDVIGSvg	0.91	0.9	4.78	0.05	4.02
	80.287–2056.97NDWI-3_vg	0.75	0.74	7.72	0.34	6.68
IPAR_InB	26.039 + 306.267NDVIGSvg+6808.693PRIcanvg	0.61	0.58	17.93	0.4	17.49
	–500.416 + 1051.142OSAVI_vg	0.33	0.31	23.22	0.09	28.59
IPAR_A7	875.05 + 36.048CTgf+6718.306PRIcanvg+509.163GNDVI-1cangf–2997.16NDWI-4cangf	0.86	0.84	17.66	0.63	15.57
	618.021 + 6935.272PRI_vg–33.644rNDVI_gf	0.66	0.63	26.31	0.24	29.22
IPAR_PM	40.181 + 12435.71PRIcanvg+1050.561SAVIcanvg–201.546SIPI-1cangf	0.8	0.78	35.33	0.11	28.02
	40.181 + 12435.71PRI_vg+1050.561SAVI_vg–201.546SIPI1_gf	0.8	0.78	35.33	0.11	28.02

Two models are presented for each trait: the first is the best combination of sensors (*bcs*) and the second the vegetation indices derived from hyperspectral measurements at the canopy level (*cVI*). bv=10 highest values for each trait.

Abbreviations: E40InB=40 d after emergence to initiation of booting period; InBA7=initiation of booting to 7 d after anthesis period; preGF=pre-grain-filling period (40 d after emergence to 7 d after anthesis); GF=grain-filling period (7 d after anthesis to physiological maturity); total=crop cycle; E40=40 d after emergence; InB=initiation of booting; A7=7 d after anthesis; PM=physiological maturity; RMSE, root mean square error; can=measurement at canopy level; FL=measurement at the flag leaf; SL=measurement at the second leaf; TL=measurement at the third leaf.

IPAR_A7_*bcs* predictions were made using CTgf, PRIvg, GNDVI-1gf, and NDWI-4gf, with *R*^2^=0.86 and RMSE=17.66 MJ m^−2^. Two of these remote sensing traits are related to canopy water content, transpiration, and plant water uptake (CT and NDWI-4) and one is related to LAI and canopy greenness (GNDVI-1) (Table 3; Fig. 1). Predictions with *cVI* were made using the photochemical reflectance index (PRI) from the vegetative period and rNDVI from the grain-filling period; model statistics were *R*^2^=0.66 and RMSE=26.31 MJ m^−2^. PLSR predictions were better than *cVI* with RMSE=24.99 MJ m^−2^ and *R*^2^=0.7 (Fig. 1).

IPAR_PM_*bcs* and IPAR_PM_*cVI* predictions were made using the same remote sensing traits, PRIvg, SAVIvg, and SIPI-1gf; *R*^2^=0.8 and RMSE=35.33 MJ m^−2^ (Table 3). PLSR predictions at physiological maturity performed the best in comparison with the other growth stages for this method, with *R*^2^=0.8 and RMSE=32.94 MJ m^−2^. IPAR_PM can certainly be predicted by any of the three methods proposed here, and similar results are obtained (Fig. 1).

### Biomass

**Fig. 2.**
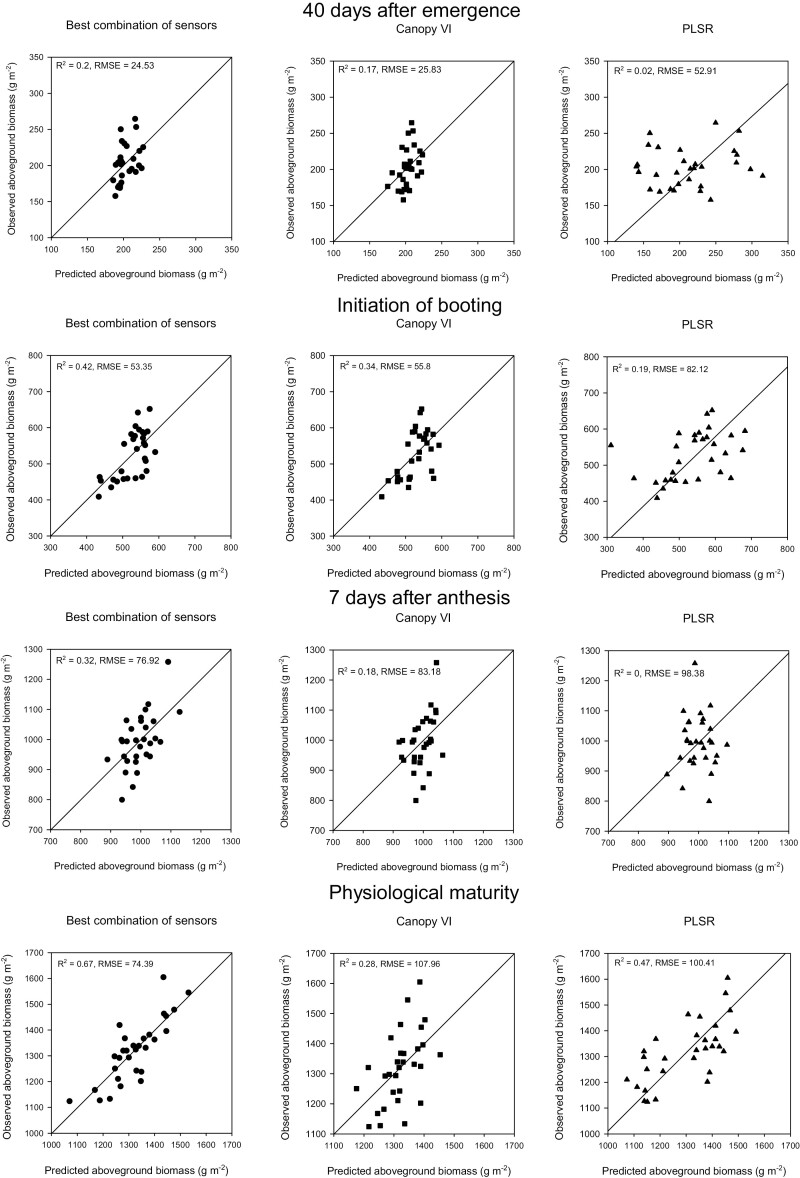
Aboveground biomass predictions with the different approaches used. Left panels represent predictions using the best combination of sensors (*bcs*), middle panels are predictions using vegetation indices derived from canopy reflectance (*cVI*), and the right panels represent predictions made with partial least squares regression (PLSR). Data points represent the genotype-adjusted means of the eight genotypes studied in Y1 and the 11 genotypes studied in Y2 and Y3 (*n*=30).

The best estimation of BM_E40_*bcs* resulted from the linear model using *J*_max_ measured in the third leaf during the vegetative period (JTLvg), with *R*^2^=0.2 and RMSE=24.53 g m^−2^, whereas the linear combination of WI, NDVI, and SAVI from the vegetative growth period resulted in the best estimations for BM_E40_*cVI*, with *R*^2^=0.17 and RMSE=25.83 g m^−2^ (Table 3). PLSR predictions at this growth stage performed less well compared with the other methods, with *R*^2^=0.02 and RMSE=52.91 g m^−2^ (Fig. 2). The use of leaf reflectance measurements to predict biomass at this growth stage performed better than predictions using canopy reflectance.

BM_InB_*bcs* was predicted using a combination of VIs measured above the canopy (NDWI-4canvg), flag leaf (GIFLvg), and third leaf (TCARITLvg). The model performance was *R*^2^=0.42 and RMSE=53.35 g m^−2^ (Table 3; Fig. 2). BM_InB_*cVI* predictions were made with NDWI-4 and TCARI measured at the vegetative period (*R*^2^=0.34, RMSE=55.8 g m^−2^). PLSR predictions were the worst of the three methods at GS41, having lower accuracy and the highest error with *R*^2^=0.19 and RMSE=82.12 g m^−2^ (Fig. 2).

BM_A7_*bcs* was predicted using PRI measured above the canopy during the vegetative period and in the flag leaf during the grain-filling period (PRIcanvg, PRIFLgf). Model performance was *R*^2^=0.32 and RMSE=76.92 g m^−2^ (Fig. 2). BM_A7_*cVI* was predicted using PRI from the vegetative period (PRIcanvg). Predictions were less accurate (*R*^2^=0.18, RMSE=83.18 g m^−2^) compared with *bcs*, but it was noteworthy that for both linear methods PRI was the common index used (Fig. 2; Table 3) and PLSR predictions performed less well compared with the other methods (*R*^2^=0, RMSE=98.38 g m^−2^) (Fig. 2).

BM_PM_*bcs* predictions were the most accurate of the three methods at physiological maturity, with *R*^2^=0.67 and RMSE=74.39 g m^−2^ (Table 3). BM_PM_*cVI* was predicted using pigment indices; these predictions were the least accurate of this growth stage for any method, with *R*^2^=0.28 and RMSE=107.96 g m^−2^. PLSR predictions were more accurate than *cVI*, with *R*^2^=0.47 and RMSE=100.41 g m^−2^ (Fig. 2).

### Radiation use efficiency

**Fig. 3.**
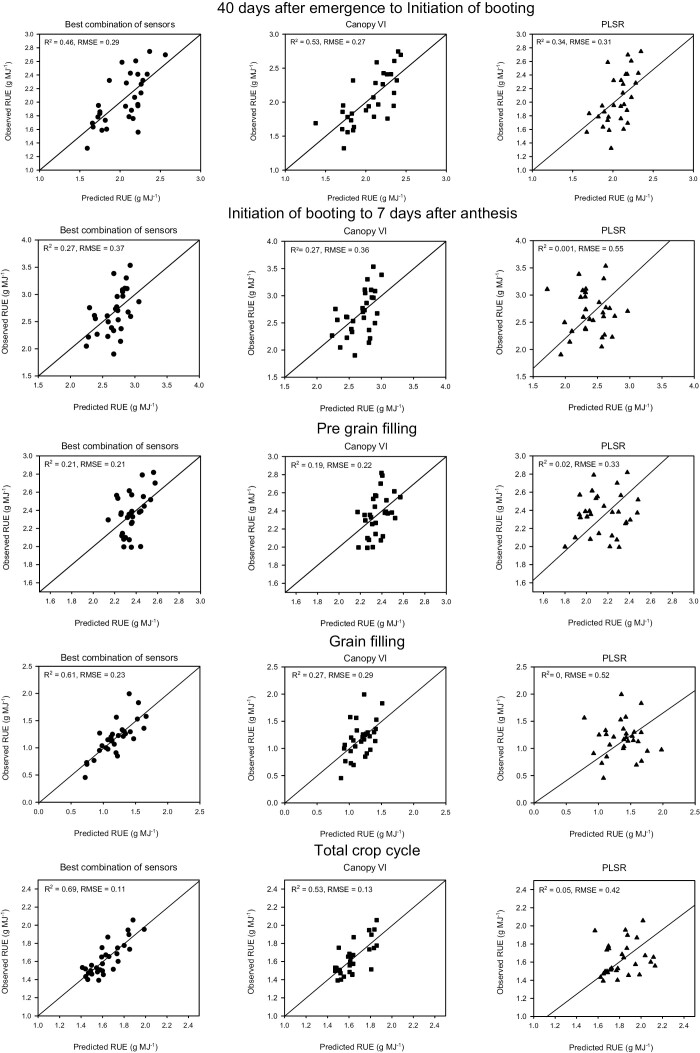
Radiation use efficiency predictions with the different approaches used. Right panels represent predictions using the best combination of sensors (*bcs*), middle panels are predictions using vegetation indices with canopy reflectance (*cVI*), and the left panels represents predictions made with partial least squares regression (PLSR). Data points represent the genotype-adjusted means of the eight genotypes studied in Y1 and the 11 genotypes studied in Y2 and Y3 (*n*=30).

From 40 DAE to initiation of booting (RUE_E40InB_*bcs*) was predicted using water and chlorophyll indices (Table 3); predictions with this method at this growth stage were less accurate in comparison with the other methods, *R*^2^=0.29 and RMSE=0.46 g MJ^−1^ (Fig. 3). RUE_E40InB_*cVI* predictions were the best at this growth stage (*R*^2^=0.53 and RMSE 0.27 g MJ^−1^. Vegetation indices used for this method were related to chlorophyll (PSSRbvg) and water content (WIvg). The PLSR model performed better than *bcs* at this growth stage (*R*^2^=0.34, RMSE=0.31 g MJ^−1^) (Fig. 3), but in general in all the traits predicted in this study PLSR modelling produced less accurate results compared with *bcs* or *cVI* models (Fig. 3; Table 3).

RUE_InBA7_*bcs* was predicted using NDWI-3 measured above the canopy, and EVI and TCARI_705_ at the third leaf (NDWI-3canvg, EVITLvg, and TCARI_705_TLvg). RUE_InBA7_*cVI* was predicted using NDWI-3 and EVI measured at the vegetative period. Both models performed the same, with *R*^2^=0.27 and RMSE=0.37 g MJ^−1^ (Table 3), and were better compared with PLSR estimations, *R*^2^=0 and RMSE=0.55 g MJ^−1^ (Fig. 3).

RUE_preGF_*bcs* was predicted using the chlorophyll content of the third leaf measured with a SPAD meter (Table 3). The model estimations with this method resulted in poor estimations, with *R*^2^=0.21 and RMSE=0.21 g MJ^−1^ (Fig. 3), but in return this model is the easiest to build as it only uses measurements from a sensor which is very easy to deploy in the field. RUE_preGF_*cVI* model performance was similar to *bcs* (Table 3), with *R*^2^=0.19 and RMSE=0.22 g MJ^−1^. All RUE predictions with the *bcs* method at the vegetative period (RUE_E40InB, RUE_InBA7, and RUE_preGF) were predicted with traits related to chlorophyll content in the bottom of the canopy. PLSR estimations were the worst of the three methods, with *R*^2^=0.02 and RMSE=0.33 g MJ^−1^ (Fig. 3).

RUE_GF_*bcs* estimations were the best, with *R*^2^=0.61 and RMSE=0.23 g MJ^−1^, but it was also the model that used most variables, which can reduce the applicability in field conditions (Table 3). RUE_GF_*cVI* estimations were outperformed by the *bcs* model, but we found a trend at grain filling where VIs related to chlorophyll content and gas exchange were used to predict IPAR and biomass (Table 3). PLSR predictions at grain filling were the worst for any model at any given growth stage, with *R*^2^=0 and RMSE=0.52 g MJ^−1^ (Fig. 3).

RUE_Total_*bcs* predictions were made with NDWI-2 measured above the canopy, CUR from the second leaf measured on the vegetative stage, and NPCI from the third leaf measured during the grain-filling period (NDWI-2canvg, CURSLvg, and NPCITLgf) (Table 3). Our results show that the predictions with the *bcs* model at physiological maturity were the most accurate of any growth stages/methods used (*R*^2^=0.69, RMSE=0.11 g MJ^−1^) for RUE; in comparison, RUE_Total_*cVI* had lower accuracy in the predictions but similar RMSE (*R*^2^=0.53, RMSE=0.13 g MJ^−1^), which indicates that RUE predictions could be done more quickly just by using VIs at the canopy scale, and the results will not differ much from the *bcs* method.

## Discussion

RUE is a key trait that underpins crop productivity due to its close relationship with photosynthesis, biomass accumulation, and yield, and it is of great interest in plant physiology and crop improvement for higher yield potential (Murchie *et al.*, 2009; Reynolds *et al.*, 2012; Hubbart *et al.*, 2018; Molero *et al.*, 2019). However, its complex nature caused by the interaction of several physiological processes affecting it at different growth stages and the difficulty in screening it in large field trials has not allowed physiologists and breeders to fully implement HTPP approaches for its prediction (Furbank *et al.*, 2019). In this study, a HTPP approach is proposed and validated with ground truth data collected during three field growth cycles by combining different remote sensing techniques using hyperspectral reflectance to calculate VIs and PLSR to develop statistical models that provide the flexibility to be tested in large wheat populations in yield potential conditions. Eventually this can be extended to populations grown under different environmental conditions (e.g. heat, drought, and nutrient deficiency stresses) or in other important crops such as rice, barley, or rye.

The implementation of this methodology can drastically reduce the time and manual labour needed to measure RUE and its components. Field aboveground biomass harvests and ceptometer measurements take time and use more resources than implementing a HTPP method to assess RUE components, and there is an opportunity to reduce the experimental error caused by different people sampling in the same experiment. If the data produced with these models are coupled with UAV (unmanned aerial vehicle) RGB, multi- or hyperspectral imaging plus a pipeline for data extraction, this can shift the narrative in physiological breeding as genetic gains for this trait are not often seen due to its phenotyping bottleneck.

### Physiological mechanisms underlying the vegetation index models

**Fig. 4.**
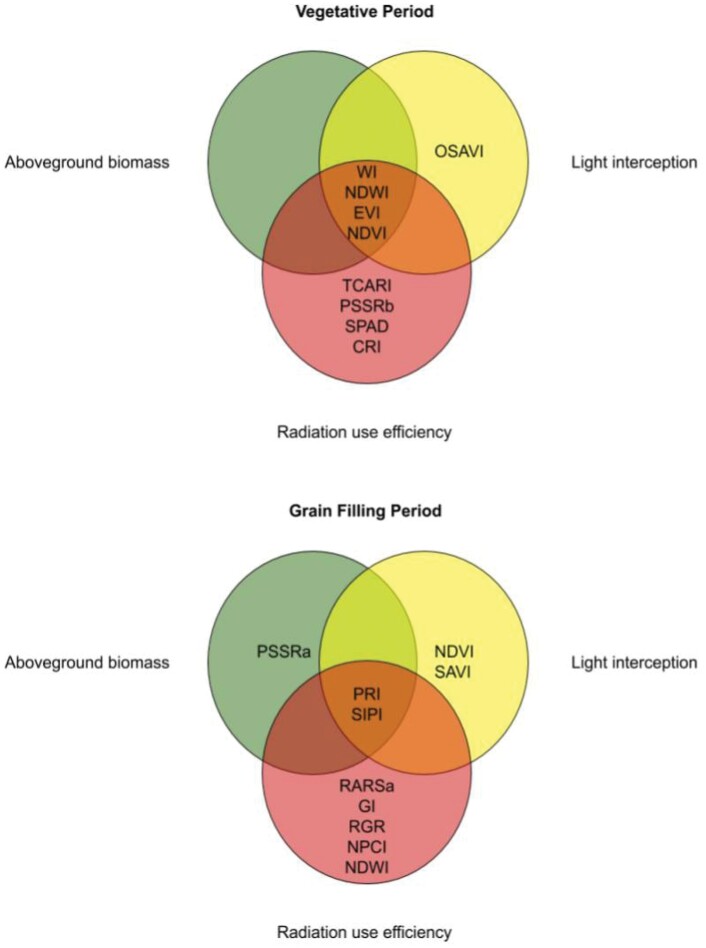
Venn diagram highlighting the correlation between remote sensing traits and aboveground biomass (green circle), light interception (yellow circle), and radiation use efficiency (red circle) during the vegetative (canopy closure to 7 d after anthesis) and grain-filling period (7 d after anthesis to physiological maturity). Indices in the middle of the diagram indicate that they can be used to predict the three traits.

Our models indicate that during the vegetative period, which encompasses the phenological stages from canopy closure to anthesis, two water indices (WI and NDWI) and two greenness indices (EVI and NDVI) were used to build models to predict RUE, biomass, or IPAR (Fig. 4). Water indices have been associated with biomass accumulation in wheat, with very strong phenotypic correlations at the vegetative stages of booting and heading (Babar *et al.*, 2006) which is within the period of our measurements for the vegetative stage. Water indices and EVI are more sensitive to variations in LAI than NDVI; this means that during the vegetative period where LAI is larger in comparison with the grain-filling stage in wheat (Calderini *et al.*, 1997), WI, NDWI, and EVI can be a better option than NDVI to predict RUE, biomass, and IPAR. Therefore, we suggest predicting RUE, biomass, and light interception using the above-mentioned VIs especially once the canopy closes and NDVI values are close to saturation (0.9). In a physiological–breeding context this becomes a problem because during the vegetative stages there are not big differences between the phenological development of different wheat genotypes, and the genotypic differences in NDVI might be negligible due to higher LAI during this growth period, while evidence indicates that water indices correlate well with biomass and, most importantly, are able to capture genotypic differences at GS41 (Babar *et al.*, 2006; Prasad *et al.*, 2009; Gutierrez *et al.*, 2010).

During the grain-filling period, the common VIs to predict RUE and its components were PRI and SIPI (structural-insensitive pigment index) (Fig. 4). Correlations between PRI and RUE indicate that if PRI increases, RUE will increase as well [higher PRI lower non-photochemical quenching (NPQ)]; this implies that there could be a source limitation or source–sink co-limitation in these genotypes at grain filling (Acreche *et al.*, 2009). PRI has been related to photosynthetic processes such as the xanthophyll cycle, NPQ, chlorophyll fluorescence, carotenoids/chlorophyll ratio, and RUE measured at leaf and ecosystem scales (Garbulsky *et al.*, 2011). NPQ plays a key role for fast annual growth plants such as rice and wheat, as it can increase productivity through biomass accumulation, and photosynthetic rates by enhancing photoprotection in high-light environments by limiting photoinhibition (Hubbart *et al.*, 2018), preventing the over-reduction of PSII and regulating the electron transport factors that can help optimize field CO_2_ assimilation (Murchie and Ruban, 2020).

Correlations of PRI with RUE have been found to be consistent across leaves, canopies, and ecosystems, with *R*^2^ ranging from 0.4 to 0.75 (Garbulsky *et al.*, 2011). In wheat, using PRI alone has been found to not be enough to predict RUE/light use efficiency (LUE) due to a drastic reduction in canopy chlorophyll content when the senescence period starts (Wu *et al.*, 2010), but our results show that using PRI combined with VI related to chlorophyll content (VARI, RARSa, and GI) and canopy senescence (SIPI) can improve the model predictions, as shown in RUE_GF_*bcs* compared with RUE_GF_*cVI* (Fig. 3; Table 3).

The activation of NPQ causes the reduction of long-term photosynthetic capacity, particularly in top and middle parts of the canopy in erect genotypes where light availability can exceed the needs of photosynthesis. In addition, leaves in the lower part of the canopy should have rapid responses to changes in light caused by the sun’s position through the day and by wind movement (Murchie and Niyogi, 2011). Efficiently disengaging photoprotective NPQ during changes from high to low light is a mechanism that has been demonstrated to increase plant biomass up to 20% in tobacco compared with plants without this ability (Kromdijk *et al.*, 2016). Additionally, slow responses of photosynthesis to increasing light could cost up to 21% of CO_2_ assimilation in wheat (Taylor and Long, 2017).

Thus, it will be possible to increase RUE by designing a new wheat ideotype with a ‘smart canopy’ for wheat with erect flag leaves to allow light penetration to lower (and usually shaded) parts of the canopy and to avoid light saturation, similarly to what has been proposed for sorghum canopies (Mantilla-Perez *et al.*, 2020). Evidence found in wheat canopies indicates that erectophile genotypes can have up to 11% higher biomass and 24% higher yields compared with planophile genotypes (Richards *et al.*, 2019); therefore, the addition of erectophile genotypes and the use of remote sensing models that correlate NPQ and PRI can become important in wheat physiological breeding to increase RUE, biomass, and yield, especially because wheat is grown under contrasting light environments across different latitudes, which leaves the door open to further increase the genetic gains of these traits.

The SIPI is correlated with the chlorophyll content and rate of senescence of the canopy (Table 2). The use of this index in our models implies that canopies that can stay greener for longer periods of time will benefit from higher biomass and IPAR accumulation, and increase RUE rates in the later stages of the crop cycle, where remobilization of nutrients to the grains, optimal N distribution through the canopy, and yield formation are critical (Foulkes and Murchie, 2011; Sinclair and Rufty, 2012). It has been suggested that developing canopies which can stay greener for longer periods of time will be one of the keystones for yield improvement in future warmer climates (Lopes and Reynolds, 2012). Although in this study models fitted better using SIPI instead of NDVI or SPAD measurements, which are usually the traits used for stay-green, this could suggest that VIs related to chlorophyll or other pigment content could potentially be used interchangeably to score senescence which is closely correlated to IPAR (Fig. 1; Table 3).

### Partial least squares regression models

To our knowledge, this is the first study where predictions of RUE, biomass, and IPAR in field-grown wheat are made with PLSR modelling. Previous attempts to predict genetic variation in physiological traits with this method have been made mostly at the leaf scale considering only the top of the canopy leaves. Traits such as *A*_max_, *g*_s_, *V*_cmax_, and *J*_max_, have been predicted successfully, with an *R*^2^ of 0.49, 0.34, 0.74, and 0.7, respectively, in spring wheat (Silva-Pérez *et al.*, 2017); *V*_cmax_ (*R*^2^=0.89), *J*_max_ (*R*^2^=0.93), and N leaf content per mass basis (*R*^2^=0.89) on aspen and cotton (Serbin *et al.*, 2012); *V*_cmax_ (*R*^2^=0.65), N leaf content (*R*^2^=0.96), and chlorophyll content (*R*^2^=0.85) in maize (Yendrek *et al.*, 2017); and leaf dark respiration (*R*^2^=0.5–0.63), leaf N content (*R*^2^=0.91), and leaf mass per area (*R*^2^=0.75) (Coast *et al.*, 2019).

Predictions of traits mentioned above at leaf level were more accurate in comparison with our predictions of RUE or biomass where in some cases no correlation between predictions and observations were found, especially during the grain-filling period (Fig. 3, *R*^2^=0). Our hypothesis for this poor performance of PLSR models is that RUE and biomass accumulation are more complex physiological processes in the hierarchical scale of yield than gas exchange in single leaf layers or organ stoichiometry, as these two might be affected by more physiological traits occurring within the canopy, plus the effects of root physiology and biomass accumulation at different growth stages during the crop cycle. Most of the studies have used sunlit leaf measurements from the top of the canopy to upscale whole-canopy physiological processes assuming that top of the canopy leaves are representative of the whole canopy (Gara *et al.*, 2019). This is not true, especially in crop canopies where there is a very dynamic light environment caused by wind, gaps due to planting methods, poor stand establishment, lodging, pest and disease effects, or even biomass harvests, which can influence photosynthetic rates from leaves lower in the canopy, and this could reduce or boost biomass accumulation and therefore RUE (Murchie *et al.*, 2018).

**Fig. 5.**
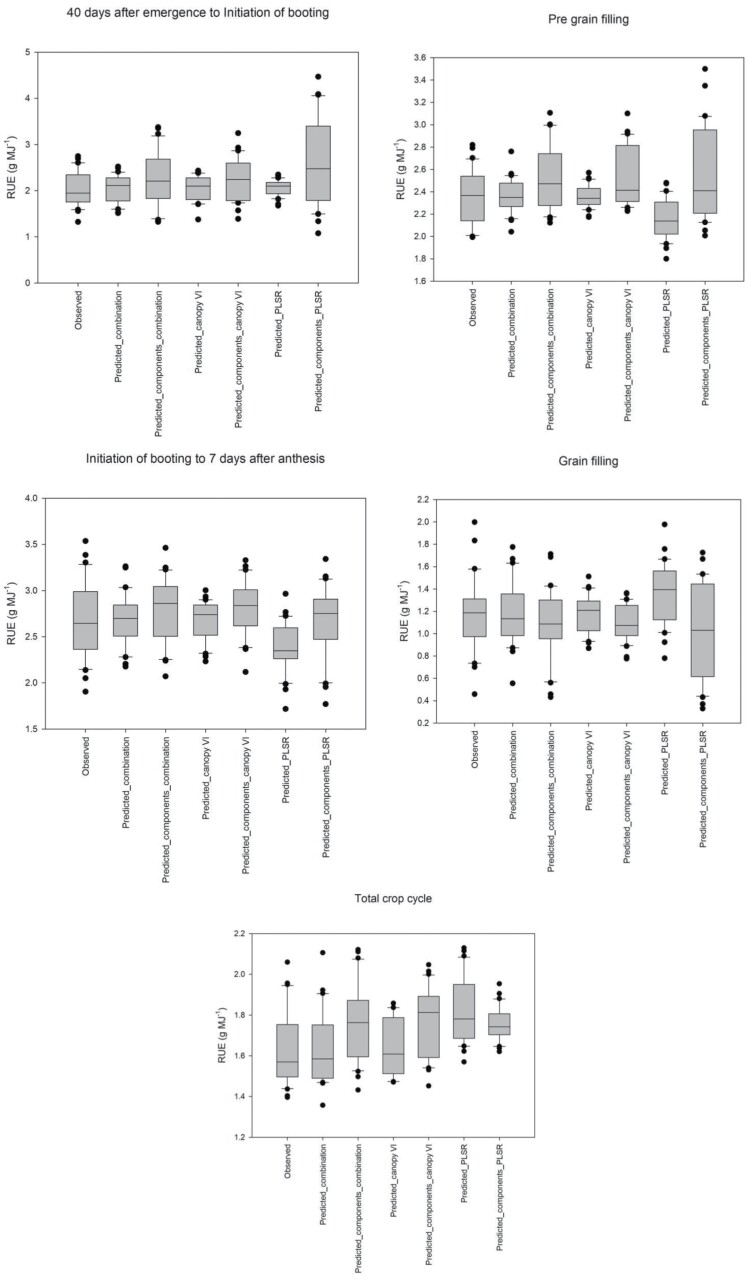
Comparison of the approaches to build the models used to predict radiation use efficiency in the different growth stages measured in the crop cycle. The *x*-axis labels from left to right represent observed values, predictions using the best combination of sensors (Predicted_combination), predictions through the estimation of RUE components (biomass and IPAR) (Predicted_components_combination), predictions using canopy vegetation indices (Predicted_canopy VI), predictions through the estimation of RUE components using canopy VI (Predicted_components_canopy VI), predictions of RUE using canopy reflectance models derived from partial least squares regression (Predicted_PLSR), and predictions of RUE through its components using PLSR (Predicted_components_PLSR).

This highlights the importance of using measurements which integrate the whole canopy instead of just the sunlit part of the canopy, and in future studies the use of punctual reflectance measurements instead of reflectance averages for different growth periods might result in better PLSR predictions for the traits presented in this study. Arguments can be made that measuring leaf reflectance and then average or integrate it from the different layers of the canopy could be used instead of measuring reflectance above the canopy to represent the canopy optical properties, but in a HTPP physiological breeding context this would take much more time in the field than collecting ground truth data, negating the benefits of the methods, and might not be worth doing as our results show that *cVI* models perform similarly to *bcs* models in most of the growth stages (Fig. 5).

Models built using VIs from the literature were the most accurate predictors of RUE, biomass, and IPAR in most of the growth stages (Fig. 5). We suggest predicting RUE directly instead of estimating it from its components since predictions of biomass and IPAR carry their own source of error, and then predicting RUE from those increases the error prediction further (Table 3). Using the models built with canopy VIs allowed us to capture the highest accuracy predicted values of RUE, biomass, and IPAR indicating genotypes that could perform the best without increasing measurement time in the field, as measuring all the leaves from the canopy could have entailed, underlining the applicability of these models in physiological breeding programmes.

### Should we rely on remote sensing for studies of growth analysis?

This is the first effort to predict RUE in a HTPP field-based physiological breeding context in wheat with data collected across three different crop cycles. The approaches to predict RUE and its components showed an acceptable level of accuracy using *bcs* or *cVI* approaches (53% in the vegetative growth stage, 61% during grain filling, and 69% considering the whole crop cycle), but we recognize that models can be improved by increasing the number of genotypes or including data from different environments. The models presented in this study have major implications for physiological breeding, as improving C fixation through RUE represents the baseline to increase crop yields. We agree that using remote sensing models cannot fully replace the collection of ground truth data, but it can considerably reduce the amount of time (i.e. from 3 d of field work and lab sample processing to 45 min measuring hyperspectral reflectance in the field) and resources spent, especially on big trials where hundreds of lines could be screened in a matter of hours and be used in quantitative trait locus (QTL) or genome-wide association studies (GWAS) to bridge the gap between phenomics and genomics. In addition, the present approach could help to predict RUE and biomass in experiments where biomass sampling is not possible due to plot size (<1 m) typically used to select plant genetic resources in pre-breeding programmes. Finally, the models built with data collected at the leaf and canopy scale in this study can be used to refine C cycle models built with satellite imagery data and increase the link between remote sensing platforms to increase our understanding of C cycle dynamics at the regional scale.

## Data Availability

The data supporting the findings in this study are available from the corresponding author, upon request.
